# Supplementary material for: A Likelihood Approach for Real-Time Calibration of Stochastic Compartmental Epidemic Models
Source: PLoS Comput Biol. 2017 Jan 17;13(1):e1005257. doi: 10.1371/journal.pcbi.1005257 (PMC5240920; doi:10.1371/journal.pcbi.1005257)
Supplement: S1 File — (TAR.GZ) [file pcbi.1005257.s014.tar.gz › HSPH_Online-SI-Revision/PF08/n1-severe/n1-severe_PF2_table0.pdf]

1	newly 3 weeks spec	27.	24.39	{5., 47.}	47.2222	0.310563	newly 3 weeks cumul	92.	81.17	{25., 154.}	42.1413	0.246007	newly 5 weeks spec	28.	19.86	{2., 37.}	50.1429	0.43242	newly 5 weeks cumul	149.	123.4	{28., 228.}	41.7181	0.26563	newly 54 weeks cumul 407. 248.51 {28., 461.} 47.3931 0.393077
2	newly 3 weeks spec	51.	31.48	{11., 58.}	47.6863	0.354837	newly 3 weeks cumul	151.	106.99	{46., 194.}	41.0927	0.252914	newly 5 weeks spec	43.	24.73	{6., 45.}	48.3953	0.441753	newly 5 weeks cumul	237.	160.33	{69., 287.}	42.5105	0.275755	newly 56 weeks cumul 398. 307.9 {110., 519.} 39.7186 0.268342
3	newly 3 weeks spec	41.	28.84	{10., 53.}	46.2927	0.314057	newly 3 weeks cumul	126.	96.67	{38., 182.}	43.9127	0.259008	newly 5 weeks spec	34.	22.71	{4., 41.}	48.5	0.382581	newly 5 weeks cumul	205.	146.5	{55., 278.}	45.2683	0.285572	newly 55 weeks cumul 432. 296.87 {78., 494.} 41.6458 0.301414
4	newly 3 weeks spec	70.	30.45	{1., 64.}	61.9571	0.656165	newly 3 weeks cumul	194.	100.6	{10., 185.}	55.0515	0.508533	newly 5 weeks spec	40.	23.66	{1., 47.}	58.4	0.575195	newly 5 weeks cumul	270.	151.04	{16., 300.}	54.2741	0.48667	newly 58 weeks cumul 393. 273.26 {30., 573.} 50.285 0.425831
5	newly 3 weeks spec	50.	25.15	{2., 57.}	57.78	0.589823	newly 3 weeks cumul	135.	83.84	{8., 177.}	53.3185	0.451901	newly 5 weeks spec	19.	18.85	{1., 45.}	70.4737	0.486077	newly 5 weeks cumul	204.	123.57	{13., 268.}	54.152	0.46152	newly 57 weeks cumul 333. 225.75 {19., 514.} 53.9309 0.452752
6	newly 3 weeks spec	37.	24.6	{7., 44.}	49.8919	0.40918	newly 3 weeks cumul	122.	83.1	{32., 153.}	47.4754	0.341584	newly 5 weeks spec	35.	20.44	{4., 37.}	53.0286	0.495192	newly 5 weeks cumul	196.	126.37	{46., 225.}	47.8724	0.367178	newly 54 weeks cumul 501. 255.13 {71., 477.} 51.8703 0.466873
7	newly 3 weeks spec	30.	23.67	{7., 40.}	41.4333	0.299912	newly 3 weeks cumul	90.	82.72	{35., 126.}	38.6667	0.21963	newly 5 weeks spec	29.	18.18	{4., 36.}	49.7931	0.420846	newly 5 weeks cumul	156.	121.21	{43., 209.}	40.6859	0.266186	newly 54 weeks cumul 252. 226.63 {67., 419.} 45.2897 0.290926
8	newly 3 weeks spec	38.	34.14	{10., 66.}	51.2105	0.380846	newly 3 weeks cumul	207.	114.06	{34., 205.}	50.599	0.435233	newly 5 weeks spec	30.	25.09	{5., 52.}	57.0333	0.455059	newly 5 weeks cumul	276.	169.2	{47., 320.}	49.6087	0.42267	newly 58 weeks cumul 301. 271.43 {58., 540.} 52.2292 0.373605
9	newly 3 weeks spec	23.	25.05	{7., 50.}	53.7826	0.248398	newly 3 weeks cumul	75.	83.9	{34., 163.}	48.6667	0.221012	newly 5 weeks spec	38.	18.65	{3., 38.}	55.7632	0.525851	newly 5 weeks cumul	137.	123.43	{43., 244.}	44.8394	0.250445	newly 54 weeks cumul 335. 230.45 {43., 415.} 46.9761 0.351796
10	newly 3 weeks spec	42.	24.97	{10., 40.}	45.5	0.30724	newly 3 weeks cumul	146.	86.01	{39., 145.}	46.0616	0.301592	newly 5 weeks spec	28.	19.6	{5., 30.}	39.3571	0.283674	newly 5 weeks cumul	205.	128.27	{53., 204.}	43.4488	0.280343	newly 54 weeks cumul 314. 253.44 {74., 427.} 37.2229 0.236603
11	newly 3 weeks spec	26.	14.13	{0., 30.}	55.9615	0.598116	newly 3 weeks cumul	86.	48.98	{8., 99.}	51.5349	0.432521	newly 5 weeks spec	24.	10.07	{0., 22.}	63.2917	0.766598	newly 5 weeks cumul	140.	71.03	{9., 148.}	54.1071	0.493109	newly 51 weeks cumul 390. 133.89 {9., 284.} 66.0744 0.714314
12	newly 3 weeks spec	37.	33.53	{7., 65.}	45.6486	0.305525	newly 3 weeks cumul	137.	117.73	{29., 205.}	38.6058	0.254961	newly 5 weeks spec	21.	23.29	{3., 45.}	59.1905	0.320058	newly 5 weeks cumul	184.	168.37	{47., 311.}	40.8098	0.239139	newly 59 weeks cumul 237. 285.81 {94., 531.} 52.5949 0.226451
13	newly 3 weeks spec	52.	26.54	{8., 49.}	52.7692	0.431287	newly 3 weeks cumul	157.	90.19	{36., 157.}	46.8217	0.358671	newly 5 weeks spec	31.	20.65	{6., 40.}	47.2581	0.388329	newly 5 weeks cumul	230.	134.97	{53., 241.}	46.6391	0.358633	newly 54 weeks cumul 413. 251.79 {73., 483.} 47.0048 0.382272
14	newly 3 weeks spec	54.	33.54	{2., 64.}	56.3333	0.527548	newly 3 weeks cumul	204.	106.49	{10., 201.}	52.1225	0.506853	newly 5 weeks spec	35.	26.36	{0., 56.}	61.7714	0.569344	newly 5 weeks cumul	282.	163.47	{15., 310.}	53.1525	0.500339	newly 59 weeks cumul 417. 291.65 {36., 578.} 49.3789 0.447612
15	newly 3 weeks spec	15.	22.52	{3., 40.}	87.0667	0.381087	newly 3 weeks cumul	83.	77.78	{17., 141.}	47.2771	0.276861	newly 5 weeks spec	28.	16.72	{1., 37.}	55.4286	0.533421	newly 5 weeks cumul	134.	114.36	{20., 217.}	45.8507	0.301685	newly 53 weeks cumul 180. 209.16 {25., 398.} 64.0778 0.338585
16	newly 3 weeks spec	53.	35.13	{6., 80.}	54.3585	0.477912	newly 3 weeks cumul	165.	107.48	{17., 224.}	48.6182	0.418483	newly 5 weeks spec	40.	28.38	{1., 62.}	60.4	0.552977	newly 5 weeks cumul	245.	167.89	{21., 354.}	51.4571	0.43167	newly 59 weeks cumul 406. 318.39 {44., 661.} 50.6084 0.414971
17	newly 3 weeks spec	52.	34.55	{8., 68.}	44.9423	0.386918	newly 3 weeks cumul	182.	117.98	{21., 212.}	42.7143	0.340423	newly 5 weeks spec	22.	25.75	{3., 57.}	73.4091	0.425545	newly 5 weeks cumul	246.	174.06	{36., 329.}	43.1789	0.333211	newly 59 weeks cumul 311. 282.47 {44., 553.} 46.209 0.30756
18	newly 3 weeks spec	25.	28.19	{1., 64.}	72.76	0.499105	newly 3 weeks cumul	80.	90.99	{7., 190.}	72.0625	0.443963	newly 5 weeks spec	24.	22.21	{0., 50.}	67.2083	0.582056	newly 5 weeks cumul	126.	138.51	{10., 306.}	68.2778	0.435427	newly 59 weeks cumul 490. 291.92 {20., 569.} 50.0939 0.514535
19	newly 3 weeks spec	33.	17.31	{4., 32.}	53.1818	0.451327	newly 3 weeks cumul	111.	61.69	{21., 109.}	48.6757	0.352108	newly 5 weeks spec	35.	12.89	{1., 24.}	64.6571	0.659998	newly 5 weeks cumul	180.	90.17	{27., 163.}	52.3167	0.401718	newly 52 weeks cumul 331. 168.71 {40., 321.} 52.1601 0.436049
20	newly 3 weeks spec	32.	19.66	{4., 37.}	50.8125	0.435233	newly 3 weeks cumul	124.	68.36	{18., 125.}	51.1774	0.400841	newly 5 weeks spec	27.	13.52	{1., 28.}	56.7407	0.583468	newly 5 weeks cumul	188.	98.46	{23., 180.}	52.6596	0.4342	newly 52 weeks cumul 258. 181.13 {27., 347.} 49.376 0.401071
21	newly 3 weeks spec	34.	32.63	{3., 62.}	59.5588	0.447019	newly 3 weeks cumul	127.	103.85	{10., 201.}	51.0157	0.405626	newly 5 weeks spec	35.	25.92	{2., 57.}	59.2	0.52865	newly 5 weeks cumul	201.	158.82	{12., 309.}	51.2438	0.411511	newly 59 weeks cumul 389. 301.74 {36., 597.} 48.8997 0.410247
22	newly 3 weeks spec	28.	25.84	{7., 45.}	44.7143	0.294131	newly 3 weeks cumul	132.	89.56	{31., 153.}	41.7879	0.294706	newly 5 weeks spec	29.	19.14	{3., 35.}	46.069	0.445659	newly 5 weeks cumul	187.	130.01	{43., 227.}	40.8503	0.296546	newly 53 weeks cumul 239. 226.48 {53., 403.} 45.6151 0.284608
23	newly 3 weeks spec	28.	14.28	{0., 30.}	62.3571	0.676732	newly 3 weeks cumul	106.	51.05	{5., 105.}	59.2736	0.597763	newly 5 weeks spec	31.	11.43	{0., 22.}	69.3871	0.831059	newly 5 weeks cumul	167.	75.85	{7., 149.}	61.6587	0.641603	newly 55 weeks cumul 324. 150.5 {8., 339.} 66.6667 0.730004
24	newly 3 weeks spec	36.	18.85	{5., 39.}	55.5278	0.439139	newly 3 weeks cumul	101.	56.68	{20., 115.}	51.2079	0.356127	newly 5 weeks spec	23.	14.65	{2., 32.}	58.3913	0.485834	newly 5 weeks cumul	158.	87.97	{31., 178.}	52.019	0.375607	newly 52 weeks cumul 238. 198.97 {39., 458.} 63.3655 0.38566
25	newly 3 weeks spec	53.	18.64	{5., 33.}	64.9057	0.615751	newly 3 weeks cumul	164.	64.29	{14., 109.}	60.9085	0.536626	newly 5 weeks spec	31.	13.24	{1., 26.}	59.8065	0.604319	newly 5 weeks cumul	226.	93.1	{20., 156.}	59.0354	0.522257	newly 50 weeks cumul 295. 167.05 {24., 324.} 52.5932 0.45241
26	newly 3 weeks spec	42.	23.96	{1., 57.}	60.	0.655374	newly 3 weeks cumul	125.	78.67	{6., 177.}	55.656	0.536485	newly 5 weeks spec	18.	17.4	{0., 43.}	73.1111	0.556191	newly 5 weeks cumul	181.	116.11	{10., 268.}	56.5359	0.53911	newly 57 weeks cumul 332. 215.92 {13., 482.} 55.4277 0.551732
27	newly 3 weeks spec	52.	24.26	{2., 58.}	61.6154	0.614961	newly 3 weeks cumul	169.	83.1	{10., 191.}	58.7456	0.568417	newly 5 weeks spec	14.	16.35	{1., 39.}	85.0714	0.519739	newly 5 weeks cumul	215.	119.63	{16., 283.}	57.7721	0.537923	newly 56 weeks cumul 229. 221.38 {19., 493.} 64.9258 0.443915
28	newly 3 weeks spec	51.	34.14	{5., 64.}	51.9216	0.464712	newly 3 weeks cumul	140.	106.86	{14., 208.}	47.8	0.378585	newly 5 weeks spec	48.	27.25	{1., 57.}	57.6458	0.616119	newly 5 weeks cumul	238.	165.3	{16., 311.}	49.2857	0.413525	newly 59 weeks cumul 446. 308.08 {31., 613.} 49.4574 0.432864
29	newly 3 weeks spec	34.	17.84	{3., 33.}	52.7059	0.451332	newly 3 weeks cumul	114.	62.23	{15., 104.}	48.4649	0.370144	newly 5 weeks spec	17.	14.02	{1., 28.}	53.1765	0.440355	newly 5 weeks cumul	151.	91.89	{20., 161.}	44.8278	0.344398	newly 52 weeks cumul 284. 172.67 {25., 336.} 49.5458 0.408468
30	newly 3 weeks spec	37.	22.91	{3., 47.}	49.4865	0.464635	newly 3 weeks cumul	120.	77.68	{16., 140.}	47.4167	0.36996	newly 5 weeks spec	28.	18.88	{1., 39.}	54.2143	0.521349	newly 5 weeks cumul	177.	117.12	{18., 228.}	47.2542	0.384284	newly 53 weeks cumul 261. 230.58 {26., 477.} 55.8467 0.402038
31	newly 3 weeks spec	31.	23.	{4., 42.}	47.8065	0.387284	newly 3 weeks cumul	87.	76.38	{20., 148.}	46.9195	0.279687	newly 5 weeks spec	16.	17.67	{1., 32.}	65.3125	0.43741	newly 5 weeks cumul	126.	114.87	{24., 226.}	46.754	0.284722	newly 54 weeks cumul 328. 226.75 {30., 432.} 47.5274 0.392507
32	newly 3 weeks spec	17.	31.92	{8., 61.}	111.529	0.350017	newly 3 weeks cumul	59.	107.13	{37., 208.}	98.8305	0.283927	newly 5 weeks spec	26.	24.91	{5., 45.}	50.4231	0.334692	newly 5 weeks cumul	103.	159.52	{51., 304.}	78.7379	0.263507	newly 56 weeks cumul 357. 307.53 {68., 504.} 41.4146 0.269818
33	newly 3 weeks spec	51.	35.24	{5., 75.}	54.5098	0.437672	newly 3 weeks cumul	172.	113.41	{17., 233.}	48.0756	0.394938	newly 5 weeks spec	50.	25.79	{4., 58.}	59.3	0.544525	newly 5 weeks cumul	284.	170.19	{26., 352.}	51.4965	0.434175	newly 59 weeks cumul 418. 289.71 {40., 570.} 47.4426 0.391064
34	newly 3 weeks spec	13.	13.8	{3., 29.}	57.2308	0.338581	newly 3 weeks cumul	56.	48.39	{18., 93.}	42.9464	0.259579	newly 5 weeks spec	29.	10.22	{2., 21.}	65.5172	0.660529	newly 5 weeks cumul	105.	70.55	{20., 128.}	44.7905	0.319417	newly 51 weeks cumul 255. 132.25 {24., 289.} 57.4 0.487087
35	newly 3 weeks spec	56.	35.08	{5., 63.}	47.4286	0.389119	newly 3 weeks cumul	196.	120.92	{18., 216.}	43.7857	0.344559	newly 5 weeks spec	34.	26.3	{4., 61.}	52.5294	0.405009	newly 5 weeks cumul	269.	177.35	{38., 330.}	43.9145	0.33498	newly 59 weeks cumul 333. 298.43 {56., 580.} 44.1952 0.273693
36	newly 3 weeks spec	35.	35.46	{10., 60.}	47.0857	0.267646	newly 3 weeks cumul	137.	126.5	{35., 200.}	39.9416	0.229511	newly 5 weeks spec	9.	22.74	{4., 48.}	173.111	0.444103	newly 5 weeks cumul	166.	176.4	{46., 297.}	46.2169	0.232386	newly 60 weeks cumul 168. 270.7 {62., 494.} 86.1071 0.296103
37	newly 3 weeks spec	30.	14.21	{3., 28.}	55.3667	0.525901	newly 3 weeks cumul	103.	49.92	{12., 91.}	53.9223	0.45905	newly 5 weeks spec	13.	10.27	{1., 23.}	56.5385	0.464371	newly 5 weeks cumul	136.	72.99	{19., 132.}	52.0662	0.44237	newly 50 weeks cumul 294. 126.84 {23., 265.} 61.7211 0.582028
38	newly 3 weeks spec	36.	30.98	{4., 64.}	49.2222	0.405589	newly 3 weeks cumul	146.	111.01	{17., 208.}	44.0616	0.345286	newly 5 weeks spec	22.	22.53	{4., 42.}	63.8636	0.413104	newly 5 weeks cumul	199.	160.65	{23., 320.}	46.3065	0.348297	newly 58 weeks cumul 228. 250.66 {52., 479.} 57.4912 0.333344
39	newly 3 weeks spec	27.	24.37	{7., 41.}	41.5185	0.26488	newly 3 weeks cumul	108.	81.09	{27., 144.}	39.9537	0.261755	newly 5 weeks spec	28.	17.98	{2., 35.}	45.4286	0.42968	newly 5 weeks cumul	158.	119.59	{35., 198.}	38.7025	0.262077	newly 54 weeks cumul 281. 226.18 {44., 379.} 39.6797 0.286607
40	newly 3 weeks spec	44.	32.92	{2., 68.}	50.5	0.493179	newly 3 weeks cumul	123.	112.42	{15., 200.}	51.6098	0.376718	newly 5 weeks spec	23.	25.5	{1., 60.}	74.9565	0.531424	newly 5 weeks cumul	179.	166.54	{21., 315.}	53.9888	0.394225	newly 60 weeks cumul 274. 292.02 {32., 594.} 59.8467 0.395919
41	newly 3 weeks spec	39.	34.36	{8., 64.}	54.4103	0.335608	newly 3 weeks cumul	166.	115.71	{29., 211.}	48.4639	0.327335	newly 5 weeks spec	19.	24.84	{4., 53.}	80.4211	0.363561	newly 5 weeks cumul	210.	170.38	{44., 321.}	49.3333	0.306488	newly 57 weeks cumul 257. 277.55 {74., 545.} 59.8016 0.29807
42	newly 3 weeks spec	38.	22.78	{2., 54.}	60.6316	0.585825	newly 3 weeks cumul	107.	74.36	{7., 169.}	57.4953	0.47872	newly 5 weeks spec	31.	18.29	{0., 45.}	64.4194	0.65415	newly 5 weeks cumul	165.	112.52	{11., 268.}	57.9879	0.489318	newly 57 weeks cumul 391. 229.82 {19., 514.} 57.7033 0.55713
43	newly 3 weeks spec	32.	13.78	{1., 27.}	58.875	0.611572	newly 3 weeks cumul	103.	47.12	{9., 85.}	55.534	0.51588	newly 5 weeks spec	12.	9.64	{0., 21.}	59.5	0.547427	newly 5 weeks cumul	138.	68.06	{9., 119.}	53.2464	0.502575	newly 50 weeks cumul 246. 125.47 {12., 228.} 57.4024 0.557933
44	newly 3 weeks spec	38.	31.29	{1., 67.}	58.2368	0.474596	newly 3 weeks cumul	148.	100.55	{10., 205.}	51.3176	0.43013	newly 5 weeks spec	25.	24.3	{2., 52.}	65.36	0.44875	newly 5 weeks cumul	218.	151.92	{16., 310.}	53.2477	0.428191	newly 58 weeks cumul 302. 277.29 {34., 591.} 56.8775 0.388922
45	newly 3 weeks spec	39.	17.45	{1., 42.}	62.8974	0.67689	newly 3 weeks cumul	137.	63.32	{8., 139.}	60.365	0.568417	newly 5 weeks spec	24.	13.51	{0., 30.}	61.2083	0.645924	newly 5 weeks cumul	197.	92.34	{12., 210.}	60.599	0.581008	newly 55 weeks cumul 330. 161.96 {15., 391.} 61.6424 0.618053
46	newly 3 weeks spec	24.	22.18	{6., 39.}	48.5833	0.276584	newly 3 weeks cumul	77.	74.67	{24., 134.}	45.7013	0.243936	newly 5 weeks spec	26.	16.62	{1., 32.}	51.6154	0.480441	newly 5 weeks cumul	128.	111.42	{29., 190.}	44.2188	0.270207	newly 54 weeks cumul 420. 211.12 {33., 396.} 53.1476 0.46959
47	newly 3 weeks spec	53.	38.42	{9., 68.}	42.3774	0.343058	newly 3 weeks cumul	225.	131.93	{29., 235.}	45.7289	0.371459	newly 5 weeks spec	15.	28.87	{5., 54.}	118.333	0.408203	newly 5 weeks cumul	267.	192.41	{50., 347.}	39.9213	0.303359	newly 60 weeks cumul 272. 347.56 {104., 589.} 57.375 0.26789
48	newly 3 weeks spec	21.	12.63	{0., 30.}	63.9524	0.695628	newly 3 weeks cumul	100.	43.84	{1., 98.}	61.74	0.712837	newly 5 weeks spec	11.	10.17	{0., 28.}	84.2727	0.741295	newly 5 weeks cumul	125.	64.85	{1., 154.}	60.04	0.692164	newly 54 weeks cumul 216. 124.21 {1., 327.} 68.3565 0.775578
49	newly 3 weeks spec	40.	30.33	{2., 65.}	53.775	0.445292	newly 3 weeks cumul	144.	103.59	{15., 204.}	47.8819	0.35058	newly 5 weeks spec	31.	21.74	{1., 42.}	57.9355	0.519903	newly 5 weeks cumul	205.	152.3	{23., 284.}	50.5171	0.366656	newly 58 weeks cumul 301. 248.3 {44., 474.} 50.0864 0.355751
50	newly 3 weeks spec	29.	33.72	{13., 63.}	53.1034	0.231512	newly 3 weeks cumul	126.	109.21	{49., 205.}	41.5794	0.224704	newly 5 weeks spec	18.	24.95	{7., 47.}	68.7222	0.289135	newly 5 weeks cumul	161.	163.79	{74., 293.}	41.8199	0.199859	newly 56 weeks cumul 315. 314.84 {121., 542.} 42.0508 0.226481
